# A New Angular Light Scattering Measurement of Particulate Matter Mass Concentration for Homogeneous Spherical Particles

**DOI:** 10.3390/s19102243

**Published:** 2019-05-15

**Authors:** Dong Chen, Xiaowei Liu, Jinke Han, Meng Jiang, Zhaofeng Wang, Jiuxin Qi

**Affiliations:** State Key Laboratory of Coal Combustion, School of Energy and Power Engineering, Huazhong University of Science and Technology, Wuhan 430074, China; chendong2012@hust.edu.cn (D.C.); jkhan@hust.edu.cn (J.H.); d201780325@hust.edu.cn (M.J.); m201871095@hust.edu.cn (Z.W.); m201871183@hust.edu.cn (J.Q.)

**Keywords:** particle size, mass concentration, real-time measurement, light scattering, ripple width

## Abstract

Under the condition of ultra-low emission for power plants, the particulate matter concentration is significantly lower than that of typical power plants a decade ago, which posed new challenges for the particulate matter monitoring of stationary emission. The monitoring of particulate matter mass concentration based on ensemble light scattering has been found affected by particle size. Thus, this study develops a method of using the scattering angular distribution to obtain the real-time particle size, and then correct the particulate matter concentration with the real-time measured particle size. In this study, a real-time aerosol concentration and particle size measurement setup is constructed with a fixed detector at the forward direction and a rotating detector. The mass concentration is measured by the fixed detector, and the particle size is measured from the intensity ratio of the two detectors. The simulations show that the particle size has power law functionality with the angular spacing of the ripple structure according to Mie theory. Four quartz aerosols with different particle size are tested during the experiment, and the particle size measured from the ripple width is compared with the mass median size measured by an electrical low pressure impactor (ELPI). Both techniques have the same measurement tendency, and the measurement deviation by the ripple width method compared with ELPI is less than 15%. Finally, the measurement error of the real-time mass concentration is reduced from 38% to 18% with correction of the simultaneously measured particle size when particle size has changed.

## 1. Introduction

Air pollution has become one of the most serious environmental issues in China, especially particulate matter pollution. The particulate matter emitted by coal-fired power plants is one of the main pollution sources [1,2,3,4,5,6]. Currently, with the increasingly rigorous emission standards [7], the accuracy of real-time measurement of particulate matter mass concentrations should be improved urgently. With the advances in sensing technology, the particle mass concentration is measured in real-time by means of optical, acoustic, electrostatic, radial, and other methods [8,9,10,11,12,13,14,15,16,17]. Among these varied methods, the light scattering method is widely applied in the continuous monitoring of particulate matter from coal-fired power plants.

The mass concentration of particulate matter measured by light scattering has been well described by the Mie scattering theory [9,18,19,20]. However, some studies have revealed that the light-scattering measurement results of the mass concentration are greatly affected by particle size. Roebuck [21] found that the real mass concentration is the same for both 3 μm particles and 5 μm particles sizes but that optically determined concentration of the former is approximately three times that of the latter. In addition, Görner [22] calculated the scattering light of 1 mg/m^3^ particles with different particle size and found the light intensity of submicron particles could be an order of magnitude higher than micron particles. Later, Chen et al. [23] experimentally demonstrated the sensitivity of mass concentration changes with particle size at any detection angle. In coal-fired power plants, the size of particles varies with time due to the coal type, combustion condition, boiler load, and precipitator performance [6,24,25]. In these cases, the particles emitted from the stack are mainly 0.1 to 10 μm in size and the mass is concentrated in the micro size range [24,26,27]. Recently, in power plants, the light-scattering measurement deviation of particulate matter mass concentration caused by unknown particle size is amended by arduous regular manual measurements, which is not convenient and adverse to accurate real-time measurements. Therefore, the particle size should be simultaneously measured when monitoring the mass concentration of particulate matter by the light scattering method.

To measure the particle size based on the scattering light, the ratio of scattering intensity at different forward scattering angles has been used to determine particle size [28]. According to the Fraunhofer diffraction, the height of the first lobe H ∝ D^4^ and the width W ∝ D^−1^, where D is the particle diameter, which indicates the information of size from the distribution of the forward scattered light [19]. But this method is only valid for particles much larger than the wavelength and not suitable for micron or smaller particle measurement if the wavelength of the incident light is visible. Kerker et al. [29] calculated the ratio of minima to maxima that occurred in the scattered light distribution and correlated them with particle size, which suggests that the particle size could be determined due to the oscillations of the angular distribution of the scattered light. Later, Godefroy et al. [14] acquired the particle size from scattering pattern analysis techniques. Recently, the oscillations of Mie scattering was experiential analyzed by C.M. Sorenson [30], and the ripple width of the oscillations is thought to be correlated with particle size via the power law. Later, the physical explanation was studied by M.J. Berg [31]. As an application, Ghosh et al. [32] made a nice fitting between the frequency of the oscillations with the particle size and successfully obtained the size of blood cells. Afterward, the cell size was retrieved by the location of ripples with the measurement of the two-dimensional angular optical scattering patterns [33]. These methods are mainly applied in the biological cell measurements, which exhibit more uniform particle size, and the measurement process takes place in the stable liquid phase. However, if these methods are used in industry aerosol measurements, such as the particles emitted from power plants, the particle polydispersity and real-time measurement in aerosol must be further studied and tested. Given that mixing particles with different sizes will increase the complexity of the distributions of the scattered light and the real-time measurement in aerosol may affect the stability, few studies have been reported.

In this manuscript, the measurement of particle size from the ripple width of the angular distribution of scattered light will be discussed based on the Mie scattering calculations. In addition, a real-time aerosol concentration coupled with particle size measurement setup with the light scattering method is built with a fixed detector and a rotating detector, which is designed to measure the mass concentration by the fixed detector and particle size by the ratio of the two detectors simultaneously. Four quartz aerosols with different particle size are tested during the experiment, and the mass concentration with particle size was measured simultaneously by the light scattering method. This is the first report to our knowledge in which angular ripple widths are applied to measure the aerosol particle size, especially for moderately polydisperse aerosols. Furthermore, the real-time particle size will be compared with the results measured by electrical low pressure impactor (ELPI). Finally, the real-time mass concentration modified by the simultaneously particle size will be discussed.

## 2. Methods and Calculations

### 2.1. Calculation of Particle Size from the Scattering Ripple Width

The intensity of scattering light distribution can be calculated by Equation (1) according to the Mie theory [18,34]. Here, the *λ* and *I*_0_ represent the wavelength and intensity of the incident natural light, respectively, *r* is the distance of the scattering volume center to the detector, *i*_1_ and *i*_2_ are the vertical and horizontal intensity function of the scattered light, respectively, which depends on the size coefficient *α*, refraction index m, and scattering angle *θ*. The relation between mass concentration *C_m_* with the aerosol scattering light *I_t_*(*θ*) can be described by Equation (2), where *I*_0_ is the intensity of incident light and *V*(*θ*) is the scattering volume. In addition, the angular distribution of the scattering light can be described by I(θ)/I(45°) at a reference of 45 degrees according to Equation (3). The intensity functions can be calculated according to the theory by V. D. Hulst [35] and the later MATLAB^®^ functions by C. Mätzler [36] as follows:

(1) $$I(\theta )=\frac{\lambda^{2}I_{0}}{8\pi^{2}r^{2}}\left[i_{1}(D,\lambda ,m,\theta )+i_{2}(D,\lambda ,m,\theta )\right]$$

(2) $$\frac{I_{t}(\theta )}{C_{m}}=I_{0}\cdot \frac{1}{\rho }\cdot \frac{3}{4\pi^{3}r^{2}}\cdot \frac{\lambda^{2}}{D^{3}}\cdot V(\theta )\cdot \left[i_{1}(D,\lambda ,m,\theta )+i_{2}(D,\lambda ,m,\theta )\right]$$

(3) $$\frac{I_{t}(\theta )}{I_{t}(45{}^{\circ})}=\frac{\left[i_{1}(\theta )+i_{2}(\theta )\right]}{\left[i_{1}(45{}^{\circ})+i_{2}(45{}^{\circ})\right]}$$

(4) $$\Delta \theta =\sum_{1}^{n}\Delta \theta_{k}/n$$

**Figure 1:**
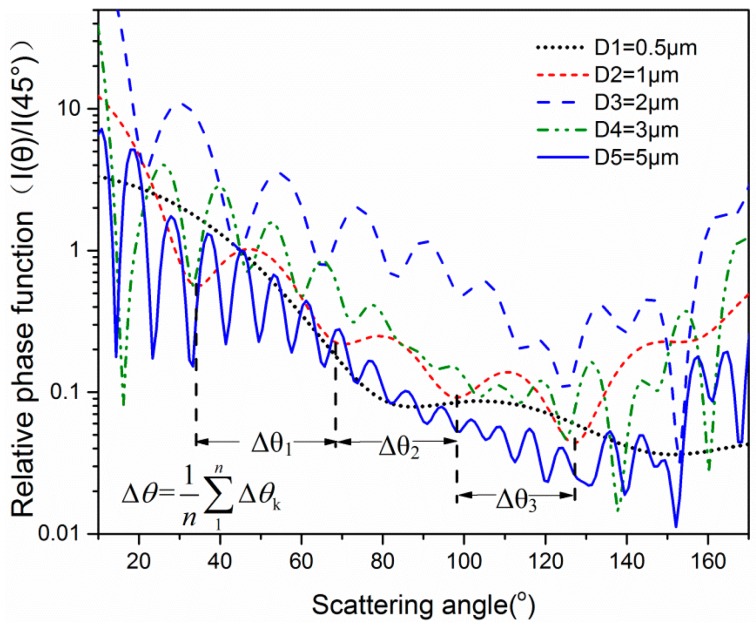
The calculated relative phase function *I*(*θ*)/*I*(45°) for different quartz particle sizes.

**Figure 2:**
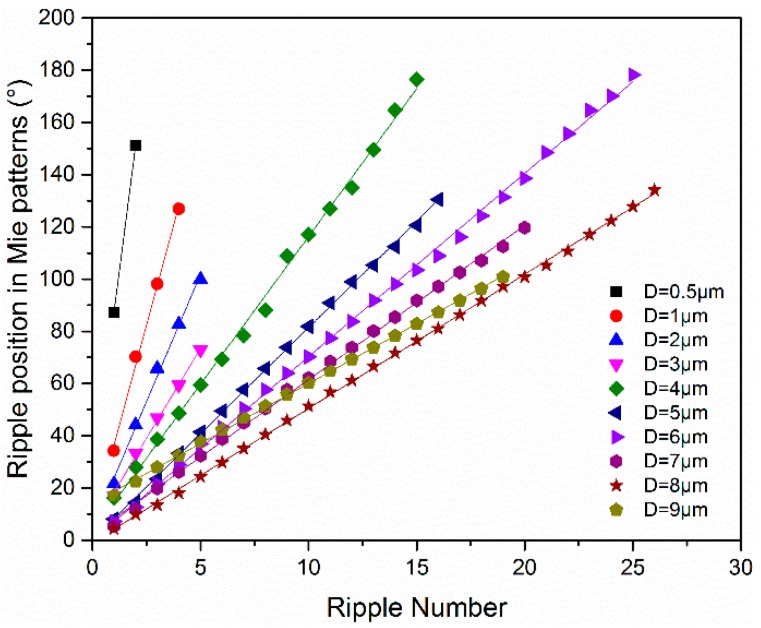
The calculated ripple position with the ripple number for the different sized quartz particles.

The calculated results of I(θ)/I(45°) versus scattering angle at different particle size are shown in Figure 1. In these cases, the wavelength of the incident light is set as 633 nm, which is consistent with the experiment. As noted in Figure 1, the oscillation of curves intensifies as the particle size increase. The ripple width can be defined as the distance between the adjacent minima of the intensity ratio. For example, for the phase function of a particle with the size of 1 μm, the first ripple width is *Δθ*_1_, and the kth ripple width is *Δθ*_k_. Thus, the average ripple width can be described by Equation (4), where *n* is the ripple number. Figure 2 presents the minimum positions in Figure 1 versus the minimum number of different particle size. As seen in Figure 2, the minimum number increased with particle size under most situations, which is similar to the results of N. Ghosh [32]. In addition, the positions of the ripples are linear with the ripple number. Thus, the data points are linear fitted, and the slopes represent the average ripple width *Δθ*. Table 1 presents the slopes of the fitting lines and the fitting linearity for the particles with different sizes. The slope values decreased with particle size. The linear correlation coefficients are considerably greater than 0.99, which means that the ripple width changes minimally with the scattering angles. This phenomenon indicates that the partial ripple width can represent the average ripple width, which reduces the amount of measurement work.

**Figure 3:**
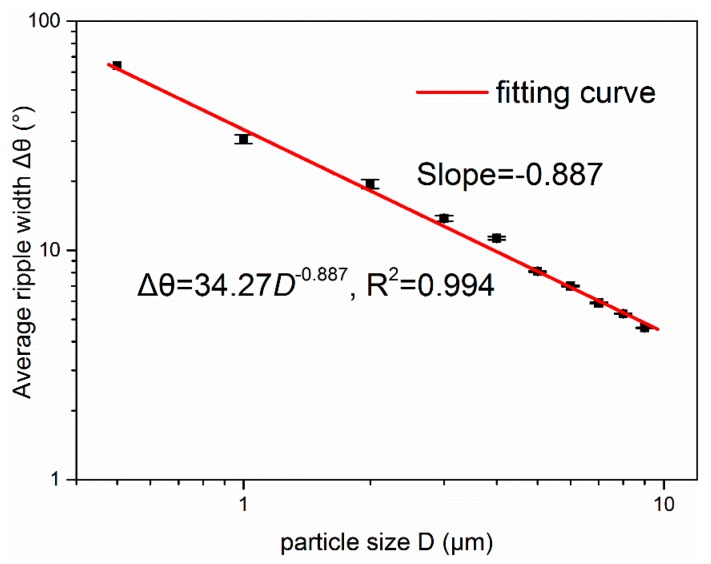
The calculated relationships between the averaged ripple width and particle size for quartz particles.

The relationship between particle size *D* and the ripple width have been discussed by some researchers [32,37]. According to C.M. Sorensen’s results [30], for the particle with a large phase shift parameter $\rho =2\pi D\lambda^{-1}\left|m-1\right|>5$, the ripple width can be experientially summarized as $\Delta \theta =\lambda D^{-1}$. It should be noted that the angles used in this paper are reported in degrees, while C.M. Sorensen reported radians. For the particles discussed in this paper, the refraction index *m* is 1.54, and for particles greater than ~1 μm in size, the phase shift parameter is greater than five. To preserve a similar law with the former experiential relationships between ripple width and particle size, a power law fit is explored, and the best fit is $\Delta \theta =34.27D^{-0.877}$. Regarding this fitting, the particle size is extended to 0.5 μm, which can also see 1 ripple from the scattering distributions. Figure 3 presents the fitting curve of the relationship between ripple width and particle size. The small difference in the exponent compared with the former experiential formula can be attributed to the lack of rigorous physical interpretation and some approximate conditions in the former studies. Moreover, the accurate calculation results from Mie theory in Figure 1 suggested that the ripple width is not exactly constant even though deviation is small and we used the average ripple width here. These findings prove that the experiential formula $\Delta \theta =\lambda D^{-1}$ is not absolutely accurate for all conditions. Thus, to obtain accurate results in this study, we used the best-fit parameter to establish the relationship between the average ripple width and particle size. The conversion formula is defined as the following Equation (5):

(5) $$D=53.76\cdot \Delta \theta^{-1.127}$$

According to the calculation results in Figure 1, the ripple is not obvious when the particle size is smaller than 0.5 μm. As to our further calculations, particles with a size larger than 10 μm still satisfy the power law fitting in Figure 3. As to the power law fitting, the increase of particle size reduces the widths of the ripples, and then the angular measurement resolution of the experiment setup is also required to improve for super-micron particles. Given the particulate matter emission from coal-fired power plants is mainly micron-sized particles and the samples in the experiment we test are PM_10_. Thus, we do not show the calculation results of super-micron particles, even though it can still be measured with this method.

### 2.2. Discussion for Particles with Different Refractive Index

**Figure 4:**
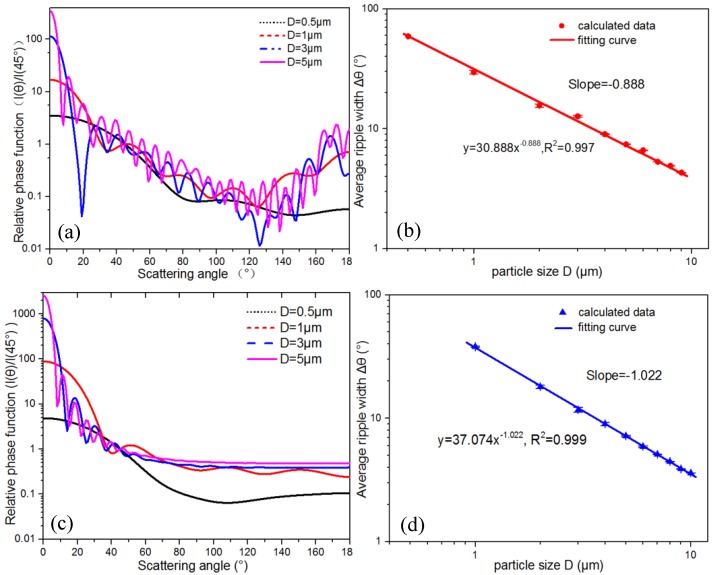
The relative phase function *I*(*θ*)/*I*(45°) with different particle size and the relationship between average ripple width and the particle size are shown in the left and right panels, respectively, for (**a**,**b**) polystyrene particles and (**c**,**d**) coal dust.

The scattering ripple characteristic is strongly dependent on the refractive index for particles with different sizes [38,39,40]. To further verify this relationship to extended particle materials, the scattering ripples of polystyrene particles and coal dust were also studied. Polystyrene is normally used as standard particle material, and coal dust has the ability of light absorption. The refraction index of polystyrene and coal dust is set as 1.58 and 2.00-0.6i, respectively [41,42]. Figure 4a,b show the scattering intensity distributions and the fitting curve of average ripple width with particle size of polystyrene particles, respectively. The scattering ripples of polystyrene particles in Figure 4a have similar patterns with the quartz particles in Figure 1 because of the similar refractive index. The best power law fit is $\Delta \theta =30.88D^{-0.888}$, and the squared correlation coefficient is 0.997. Figure 4c,d are the scattering intensity distributions and the power law fitting curve of the average ripple width with particle size for the coal dust, respectively. The decrease of ripple width with particle size for coal dust is also found in Figure 4c, but the ripples disappear at large scattering angles, different from quartz and polystyrene particles. The reason may be that the coal dust particle has an imaginary part of the refractive index, which absorbs some of the incident light and reduces the refraction and reflection of the incident light, while the diffraction light is not affected by the refractive index according to the diffraction formula. Thus the forward scatting ripples remain and the ripples in the back scattering region disappear. The ripple width in the forward scattering angles is also analyzed. The best power law fit is $\Delta \theta =37.07D^{-1.022}$, and the squared correlation coefficient is 0.999. This result suggests that the power law correlation of ripple width with particle size is still valid for the particles with a large refractive index.

**Figure 5:**
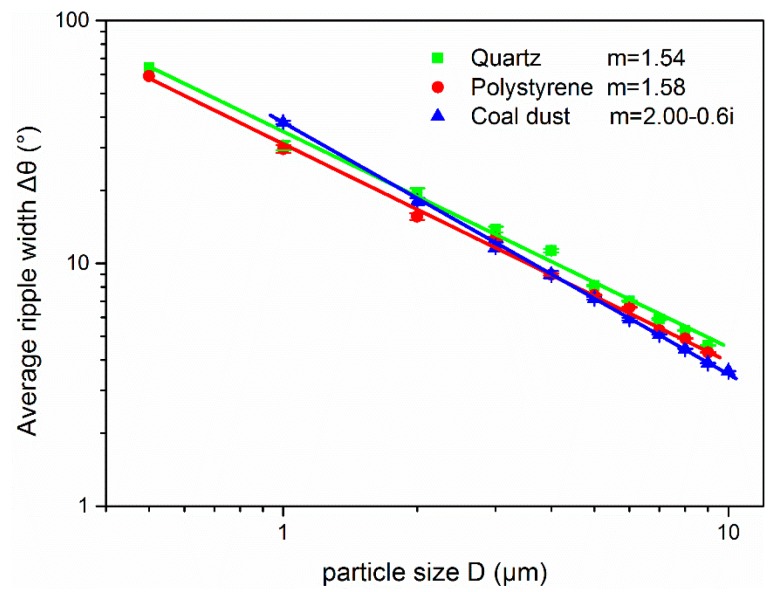
Comparison of the fitting curves between ripple width and particle size for particles with different refractive indexes.

Comparison of the fitting curves between ripple width and size for particles with different refractive indexes has also been studied, which can be seen in Figure 5. The fitting lines of the three kinds of particles are similar in the micron size range. It seems that particles with size near 3 μm a have a smaller effect on the refractive index, the average ripple width of these three 3 μm particles change within 1 degree. The measured 3 μm particle size due to Equation (5) can vary from 2.6 to 3.1 μm. This result shows that the refractive index can affect the measured particle size of this method, and smaller particle size, which has a larger ripple width, tends to have larger measurement error. 

### 2.3. Method for Particle Mass Concentration Measurement

The relationship between polydisperse particle mass concentration and the light scattering intensity of a fixed angle is described in Equation (6) according to theory by Hulst, V.D. [35] and the former study of Chen et al. [23]. The normalized particle mass size distribution *f_m_*(*D*) is described in Equation (7). Thus, for a known aerosol, the scattering light intensity is theoretically proportional to the aerosol mass concentration. Their relationship is expressed in Equation (8), where *K* is the calibration parameter of a known aerosol, *S* is the output of the detector representing scattering light intensity and *S*_0_ is the signal when the particle mass concentration is zero. In this paper, the scattering light signal for mass concentration indication is located at scattering angle of 45 degrees. The parameter *K* of an aerosol is determined by the slope of the relation curve of particle mass concentration *C_m_* and scattering light signal *S* as Equation (8).

(6) $$\frac{I_{t}(\theta )}{C_{m}}=I_{0}\cdot \frac{1}{\rho }\cdot \frac{3\lambda^{2}}{4\pi^{3}r^{2}}\cdot V(\theta )\cdot \int \frac{f_{m}(D)}{D^{3}}\left[i_{1}(D,\lambda ,m,\theta )+i_{2}(D,\lambda ,m,\theta )\right]dD$$

(7) $$1=\int_{0}^{\infty }f_{m}(D)dD$$

(8) $$C_{m}=K(S-S_{0})$$

## 3. Experimental Section

**Figure 6:**
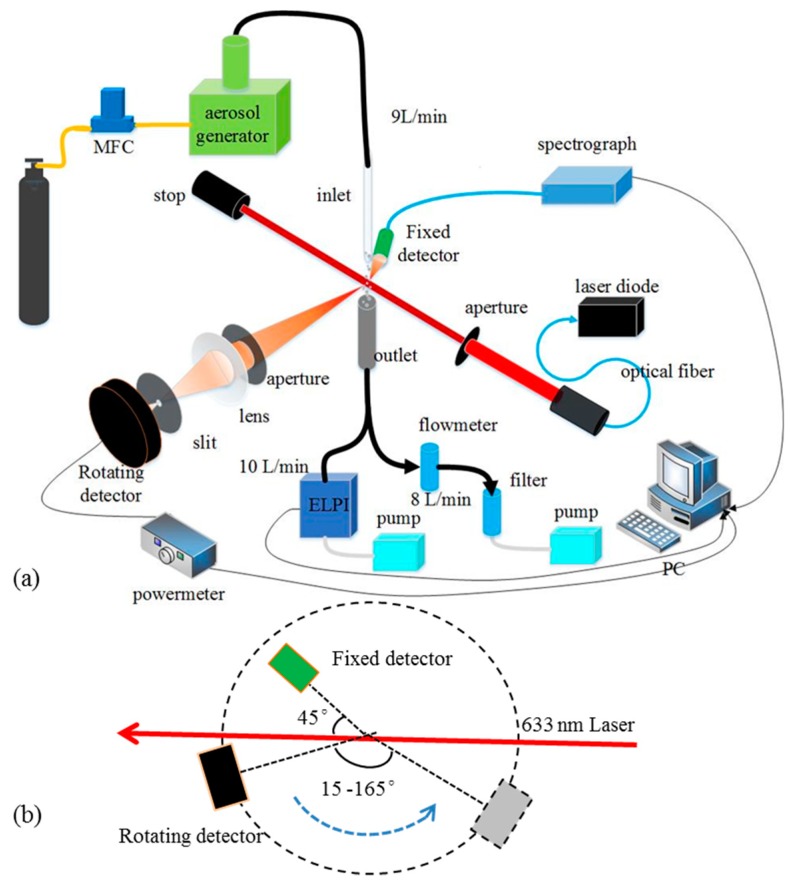
Schematic diagram of the integrated experimental setup (**a**) and the light path (**b**).

To examine the particle size measurement method and simultaneous monitoring of particulate matter mass concentration, an aerosol optical measurement platform was built. This platform mainly contains the aerosol generation–measurement system and the dual-angle scattering light detection system. The particle size is determined by angular light scattering measurements, and the mass concentration is based on the real-time response of a fixed detector. The platform illustration is shown in Figure 6.

### 3.1. Experimental Setup

As shown in Figure 6, the aerosol was generated from the Fluidized Bed Aerosol Generator (TSI 3400A) with a flow rate of 9 L/min. A cyclone of 10 μm aerodynamic cutting size was installed at the outlet of the aerosol generator. Then the generated aerosols were particulate matter with an aerodynamic size lower than 10 μm (PM_10_), which is similar to the size characterization of the particulate matter emitted from power plants after dust equipment. A 5 mm long gap was designed between the inlet and outlet, then, the aerosol went through the gap as a particle beam and was illuminated by the laser. The spot diameter of the laser at the detection volume was 2 mm and the detection volume was a 2 mm diameter and 5 mm long lying cylinder. An electronic low pressure impactor (ELPI) was connected with the outlet by a flow rate of 10 L/min to measure the mass concentration and the particle size of aerosols. The ELPI (Dekati Inc., Kangasala, Finland) divides the particles with aerodynamic size of 0.03 to 10 μm into 12 channels and measure the particles according to the particle electrification. More details can be seen in reference [43]. In addition, a bypass of 8 L/min gas was sucked from the outlet; thus, 9 L/min sheath gas came from the environment and could protect the stability of the particle beam near the detection volume. It should be noted that the environment particle concentration is two orders of magnitude lower than the aerosol sample; thus, the particles introduced from the environment can be neglected.

The dual-angle scattering light detection system was fixed on an optical table. The light source was a 633-nm diode laser (Oxxius LBX-633S, Lannion, France) with a fiber and beam collimator. The incident light was horizontally polarized, and the intensity was ~20 mW. The beam diameter after the collimator was ~6 mm, and a 2-mm diameter aperture was located in the center of the beam, which decreased the uneven Gauss beam effect to the scattering measurement. A fixed detector was located at the scattering angle of 45° and connected the spectrograph with optical fibers to measure the scattering intensity. In addition, a rotating detector system was installed on the other side of the laser beam in the detection plane, and could be rotated during the scattering angle from 15° to 165°. A common scattering angle of 45 degrees was chosen to measure the mass concentration, which has an appropriate sensitivity to meet the dynamic range of the detector when particle concentration changes from 0 to ~30 mg/Nm^3^. The angle range of rotating detector was 15° to 165° to measure the ripple space and particle size. This range was chosen because the scattering light distributions at small angles have no obvious ripple features and the angles larger than 165° are inconvenient to measure given the limitations of the platform. The semi-angles of the fixed and rotating detector for collecting scattering light were both ~5 degrees. The rotating detector system was carefully designed with a slit, lens, and apertures to limit the view of the detector at the detection volume and reduce the stray light as much as possible. In this experiment, the field of view of the detector covers all the illuminated particles at angles from 15° to 165° to keep the scattering volume constant at different angles. The rotating detector was connected with a power meter to obtain a relative scattering light intensity. The power meter (Thorlabs, PM100D) can measure the light in nanowatts with a logging interval of 1 s (3 ms integral time and 1000 ms average). The fixed detector was a 6-mm diameter fiber optic collimating probe, which was detected by a spectrograph (Ocean optics QE65Pro). The spectrograph was applied here for future multi-wavelength study. The integral time of the spectrograph was 100 ms and the record interval was 1000 ms average. Thus, the two detectors record the light intensities synchronously at 1 s. The 45° fixed detector can indicate the real-time mass concentration of the aerosols. In addition, the intensity ratio of the rotated detector with the fixed detector I(θ)/I(45°) can represent the angular distribution of the scattering light, which eliminates the effect of aerosol concentration fluctuation. Then, the angular light scattering and the average ripple widths of the scattering patterns of the aerosols can be analyzed to obtain the particle size. Of note, the response time of the particle size depends on the rotation period, and the rotation speed is precisely controlled by a stepper motor. In this paper, the rotating detector stayed on every 5 degrees for 5 s, and the total rotation duration was about 3 min. Then, we used the particle size measured in the last 3 min to correct the particle concentration for the next 3 min, which is applicable under actual situations given that particle size does not change rapidly in a few minutes.

### 3.2. Material

**Figure 7:**
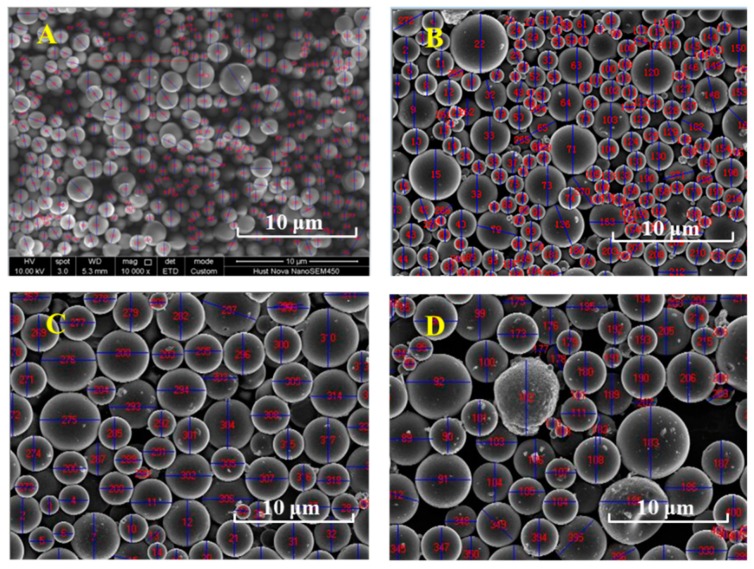
Scanning electron micrographs of the four spherical quartz aerosol samples of A, B, C and D, respectively (scale 10 μm).

**Figure 8:**
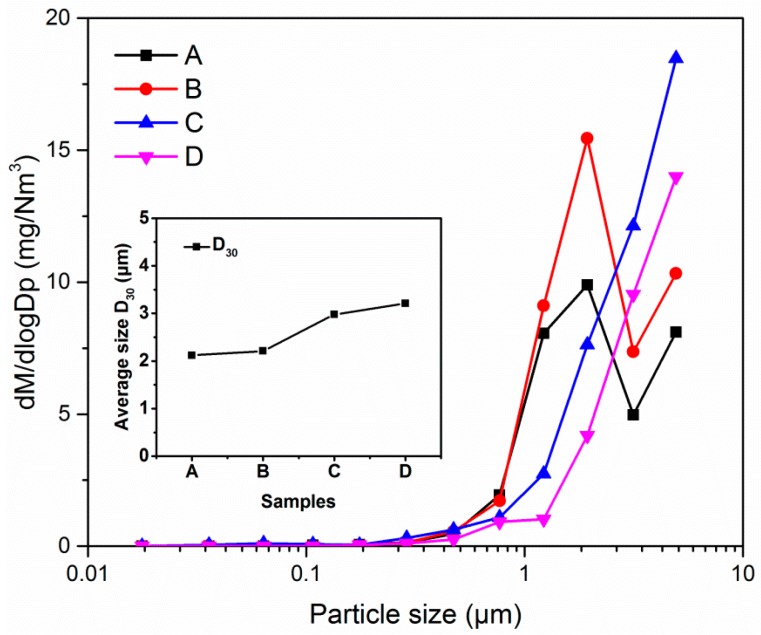
The size distributions of the four particles measured using the reference method.

To test the measurement of particle size by the ripple width; and verify the contribution of the simultaneously measured particle size on the mass concentration, four quartz particles A, B, C, and D with different sizes were chosen as the aerosol samples. Figure 7 presents the scanning electron micrograph (SEM) of the four samples. These particles are approximately spherical to eliminate the effect of particle shape. In addition, the emitted micron-sized and submicron-sized particles after the dust-removing equipment in coal-fired power plants are mostly spherical because they are mostly formed by the mechanism of volatilization and condensation. The reference instrument ELPI measured the particle size distribution and calculated the medium diameter of the particle size, which can be seen in Figure 8. Of note, the aerodynamic sizes of ELPI have been converted to geometric diameter according to the formula in reference [44], and all the diameters in this paper are geometric diameters, which is convenient for optical measurement and calculation. The results indicate that the mass medium diameters of aerosols A, B, C, and D are 2.02 μm, 2.03 μm, 2.62 μm, and 2.63 μm, respectively; however, the dispersion index is slightly different.

### 3.3. Verification of the Experimental Setup

**Figure 9:**
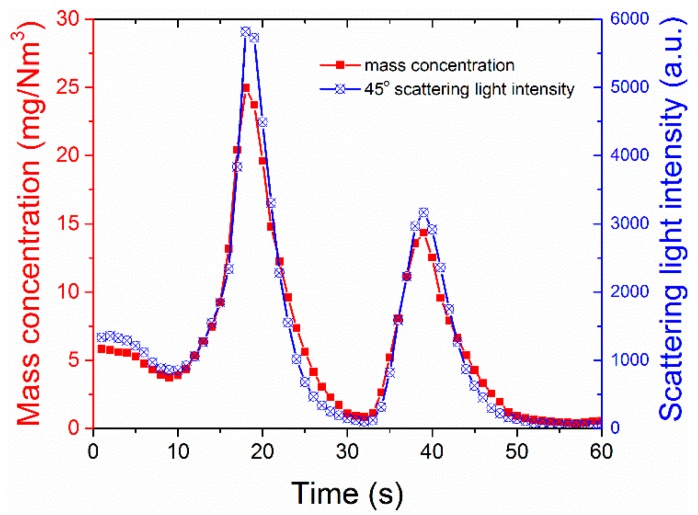
Real-time readings of mass concentration measured using the reference method and scattering light intensity during the experiment of sample A at a scattering angle of 45° within 1 min.

To verify the aerosol mass concentration measurement availability of the experimental setup, the real-time records of mass concentration measured by ELPI is compared with the scattering intensity measured from the fixed 45° detector. For example, Figure 9 represents the simultaneous readings of the two devices for aerosol B within 1 min. The real-time mass concentration of the aerosol measured by ELPI is thought to be accurate and used as a reference for the light scattering method. According to Maricq’s results [45], the mass concentration error measured by ELPI is within 20%. The mass concentration is in the range of 0 to 30 mg/Nm^3^ (milligram per cubic meter at the standard condition) and changes quickly with time, which is consistent with the emission conditions of particulate matter in industrial environments, such as power plants. These results indicate the effectiveness of the generated aerosol concentration. In addition, the transmission of the light should be greater than 60% if multiple scattering does not occur. The transmission of the light can be calculated by Equation (9) [19]. Table 2 presents the particle concentration of nine size bins greater than 0.1 μm for sample A as measured by ELPI. When the length of the optical path *L* = 0.005 m, *C_ext_* is the extinction cross-section, a function of particle size *D* and refractive index *m* = 1.54 as calculated with the Mie theory, and *n(D)* is the particle number distribution as noted in Table 2. Here we ignore the minimal contribution of the particles less than 0.1 μm. The calculation result of the transmission is *I_t_*/*I*_0_ = 99.997%, which indicates that only single scattering occurs in the experiments.

(9) $$\frac{I_{t}}{I_{0}}=\exp(-\int_{0}^{\infty }LC_{ext}n(D)dD)$$

## 4. Results and Discussion

### 4.1. Moderately Polydisperse Particle Size Measurements from Ripple Space

**Figure 10:**
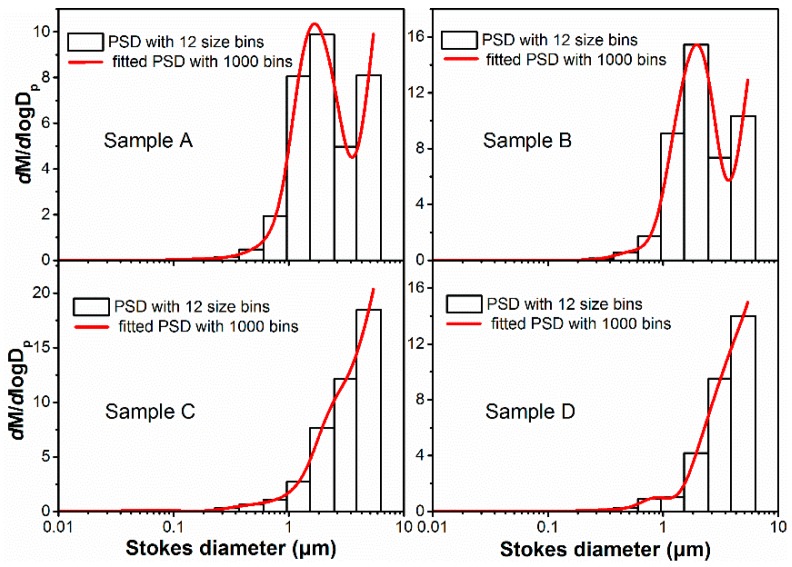
The particle size distributions (PSDs) of the four aerosol samples (A–D). (The histograms represent the measurements of the instruments with 12 size bins, and the curves represent the interpolating fitting with 1000 size bins).

**Figure 11:**
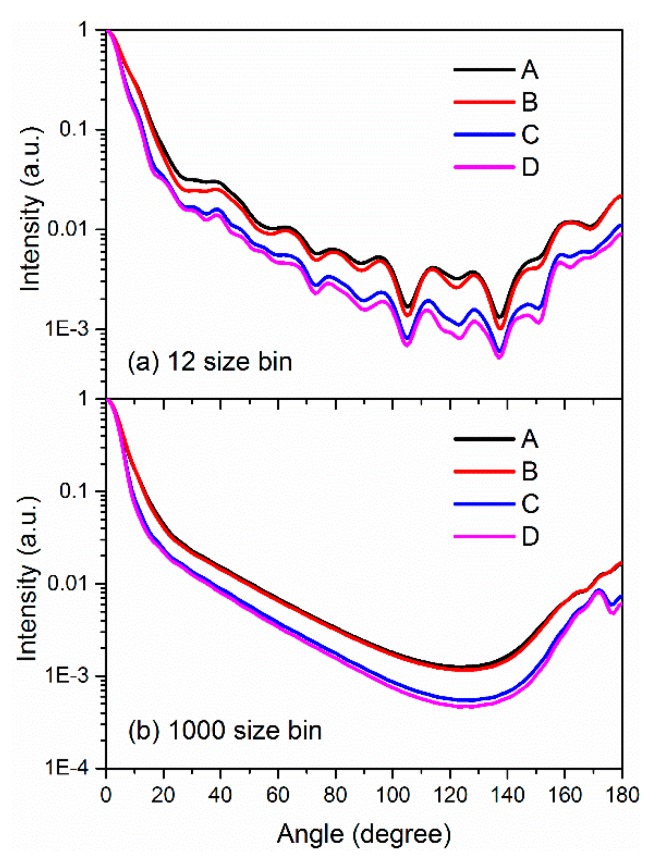
The simulated relative intensity distributions of the scattered light of the four samples with (**a**) the measured 12 size bin PSDs and (**b**) the fitting 1000 size bin PSDs.

Natural particles are mostly polydisperse and not monodisperse. Thus, the application of the particle size indication by scattering ripple width should be verified under at least moderately polydisperse particles. Figure 10 presents the particle size distributions in the experiment with different particle numbers. In Figure 10, the histograms are the ELPI measurement results with 12 size bins that represent the discontinuous of particle size distributions when the particle amount is small. The red curves represent the interpolating fitting with 1000 size bins, and the same distributions are noted with ELPI measurements. These curves represent continuous particle size distributions when the particle number is large. Given these different size distributions, the angular distributions of the scattering light of the four samples can be calculated with Equation (2). Figure 11a,b represents the simulated scattering light distributions with different particle numbers of 12 size bins and 1000 size bins for the four aerosol samples. When the particle size distribution ranged from 0.01 to 10 μm and is separated by 12 bins, obvious scattering ripples can be observed for all the four samples. When the particle number increases, and the size bins can increase to 1000, the particle size distributions are finely divided by every 0.01 μm. In addition, the calculated scattering light distributions become smooth, and the ripples disappear. Our further calculations indicate that the ripples are strongly dependent on the continuity of particle size, and the visibility of the ripples reduces with the increase of segments of the particle size distributions (PSDs) to the polydisperse particles, such as the common log-normal and Rosen–Rammler distributions. This finding is attributed to the fact that the successive sized particles have similar ripple patterns but a slight shift. When the size of the aerosol has sufficient continuity, the waning and waxing intensity of scattered light for the particles with adjacent size can wash out the ripples of the scattered light distribution for particles with a disperse PSD, leaving only the broad outline of the patterns [30]. As a result, natural aerosols, which have continuous size distributions, typically exhibit obscure scattering ripples.

However, only large particle numbers can ensure the continuity of particle size. Thus, when using the ripple width to retrieve the particle size for moderate polydisperse aerosols, limiting the scattering volume can reduce the number of particles measured at one time. In addition, reducing the particle number can increase the continuity of the particle size, which helps make distinct ripples. In our experiment, the scattering volume is 15.7 mm^3^, and the aerosol concentration is approximately 10 mg/Nm^3^. The particle number of aerosol A in the scattering volume is calculated in Table 2. According to the calculation in Table 2, when the particle mass concentration is 6.7 mg/Nm^3^, the scattering volume has approximately 145# particles larger than 0.1μm, including only 13# micron-size particles. This finding demonstrated that the particle number in the scattering volume is small and the particle size is discontinuous. In addition, the integral time of the rotating detector is ~3 ms, and the aerosol velocity is 7.64 m/s. Thus, the sampling particle number under this duration is ~10 times of that in scattering volume. With this small scattering volume design, the particles in the scattering volume are sufficiently discrete, and the scattering ripples can be prominent.

Under the high-temperature coal combustion condition, the coal-generated micron and submicron particles are mostly spherical given the mechanism of vaporization/condensation of the mineral elements in coal [24,26]. For many other particle measurement fields, particles, such as ash particles in fluidized bed furnace, sea-salt particles, soot aggregates, volcanic particles, and other dust particles in the atmosphere, exhibit an irregular shape [46,47,48,49], so the adaptability of this method to irregular particles should be taken into consideration. Recently, some research studies have assessed the scattering character of nonspherical particles with discrete dipole approximation (DDA) [50] and T-Matrix methods [51]. These studies found that the scattering characteristics of nonspherical particles were affected by both the shape and orientation, which is quite different from the spherical particle and Mie theory [52,53]. According to the Mishchenko’s results, the semi-axis ratio for spheroids can change the ripple shape and positions in the angular scattering distribution, but the random orientation of nonspherical particles can wash out the ripples [53]. Zubko [54] and Sorensen et al. [55] also found the scattering distributions are smooth even for monodisperse irregularly shaped particles. Thus, the method of using scattering ripples width to determine particle size for nonspherical is not adaptable and needs further improvement for the fade of ripples. Fortunately, it is still a simple method to obtain the size of spherical particles based on their distinct ripples, which can be applied in the measurement of the particulate matter after dust removal devices, which is mostly spherical for its formation mechanism of volatilization/ condensation [5,24].

### 4.2. Particle Size Measurement from the Scattering Ripple Space

**Figure 12:**
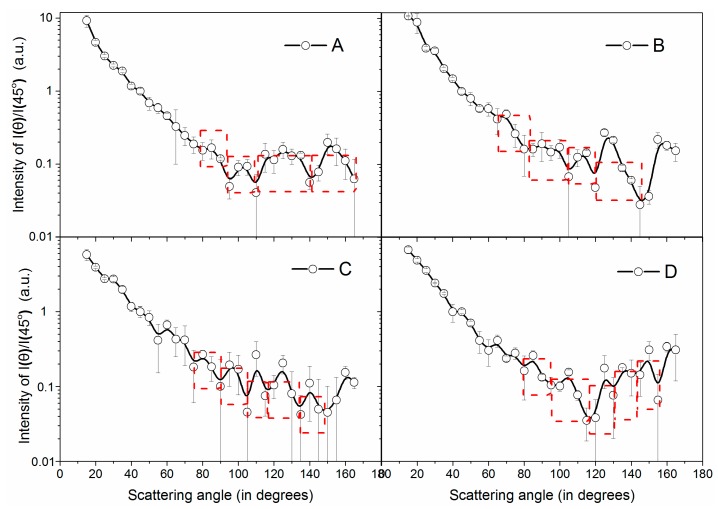
The measured angular distributions of the scattering light (45° as a reference) for the four particle samples A, B, C, and D.

In the experiment, the simultaneous intensity ratio of scattering light *I*(*θ*)/*I*(45°) measured by the rotating detector and the fixed detector was recorded during one period of rotation. Figure 12 presents the intensity ratio of *I*(*θ*)/*I*(45°) for the four particles as the scattering range ranged from 15° to 165°. The measured scattering light intensities are larger than the detection limit, and the error bar is obtained from three repeated measurements. The measurement values of two detectors are considered to be the background when the aerosol is replaced by pure gas before the aerosol measurement. The measured *I*(*θ*)/*I*(45°) indicates the angular distribution of the four particles in Figure 12, which have similar ripple characteristics with the calculated scattering patterns in Figure 1 and Figure 11a. Thus, the small scattering volume in our experiment ensures the discontinuity of the particle size and reserves the scattering ripples of the moderate polydisperse aerosols. However, the ripple at the forward scattering angles becomes weak, even though it obviously remains at the back scattering angles. This finding is attributed to the fact that the particles are modest polydisperse while the particle calculated in Figure 1 is considered absolutely monodisperse. In fact, the ripples in the forward scattering angles are more easily washed by the polydispersity because these ripples have great height and the scattering intensity decrease strongly with angles. Fortunately, for the ripples located in the right and back scattering angles, the ripples are mild, and the ripples of the dominant particles can still remain. Therefore, the ripples at large angles in Figure 12 can indicate the major particle size, which is roughly regarded as the average particle size here.

Qualitatively, the number of the ripples increase from particle A to particle D, which suggest that the average particle size increased from A to D according to the fitted line in Figure 3. In addition, despite the ripples, for the entire distribution of patterns, the angular distribution of particle A seems stable in the back scattering regions. However, the intensity of particle D has a significant increase when the scattering angle is greater than 120°. According to the Mie theory, small particles have similar right and back scattering intensities, while the back scattering increases to a greater extent than the right with the scatter size increase [18]. This result also indicates that particle D has a larger size than A.

To obtain the particle size and quantify the average ripple width of the patterns, only the obvious ripples are counted in the experiment. The results are framed by the dashed box in Figure 12. Hence, the average ripples of the four particles and the particle size can be calculated from Equation (4) and the fitting formula Equation (5). Table 3 presents the calculated particle size from the ripple width. The average particle size increases from 1.72 μm for particle A to 2.54 μm for particle D. This finding is consistent with that the particle size increase from A to D in the SEM images in Figure 7. In addition, to estimate the accuracy of the particle size measured by the ripple width, the results are compared with the mass medium diameter from the ELPI measurements. Table 3 presents the average particle size measured by ELPI and the corresponding relative error of the ripple width method. Of note, the aerodynamic sizes of ELPI have been converted to geometric diameter according to the formula provided by Mercer [44]. The size from the ELPI results also increases from A to D, and the average error of the ripple width method referring to the ELPI measurements is in the range of 3% to 15%, which indicates that the accuracy of particle size measured by ripple width is acceptable. In conclusions, the ripple width particle size measured from the angular distribution of the scattered light is suitable to indicate the average particle size of the moderately disperse aerosols.

### 4.3. Real-Time Mass Concentration Measured by the Fixed Detector

**Figure 13:**
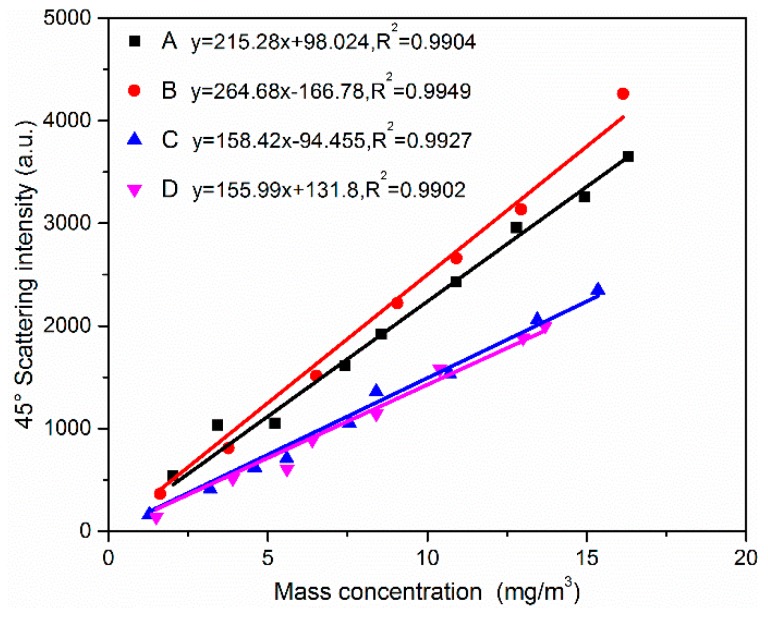
The correlation of scattering intensities measured by the fixed detector with mass concentration measured by the reference method for the particles A, B, C, and D.

The data collected by the 45° fixed detector is correlated to the ELPI mass concentration to indicate the mass concentrations of particulate matter. As noted in former studies [56,57], the scattering light intensity is generally proportional to the mass concentration for a fixed diameter. Thus, the 45° scattering light intensity was linearly fitted with the ELPI mass concentration. Figure 13 presents the correlation of the scattering intensity with the mass concentration and the fitting lines. The parameters in the fitting formula y=ax+b are presented in Figure 13 and all the correlation coefficients R^2^ are greater than 0.99. Then the mass concentration can be calculated from the scattering light intensity according to Equation (8). The relationship of the scattering light intensity with the mass concentration for the aerosol A is $I(45{}^{\circ})=2.15\times 10^{2}*C_{m}+98.02$, which means that the real-time mass concentration *C_m_* can be retrieved by $C_{m}=4.65\times 10^{-3}*\left[I(45{}^{\circ})-98.02\right]$. The intercept −0.46 suggests that the zero drift of the mass concentration by light scattering method in this setup is lower than 1 mg/Nm^3^. The slopes are similar for couples (A, B) and (C, D), which is consistent with particle size in Figure 8 given that the particle sizes are similar for (A, B) and (C, D). This result indicates that the concentration transform factor *K* of this method is strongly affected by particle size. For example, the concentration transform factor *K* changed from 3.78 × 10^−3^ for particle A to 6.31 × 10^−3^ for particle C, and the mass medium particle size measured by ELPI changes from 2.03 μm to 2.62 μm, accordingly. To further characterize the measurement error, the error of particulate matter mass concentration is described by the standard residual error of the linear regression analysis according to the following Equation (10). Here, *N* is the number of the fitting points, and C_m,t_ is the predicted concentration according to Equation (8). Then the standard error ∆*r* of mass concentration of the four samples calculated by Equation (10) are 0.53 mg/Nm^3^, 0.40 mg/Nm^3^, 0.43 mg/Nm^3^, and 2.00 mg/Nm^3^ for A, B, C, and D, respectively.

(10) $$\Delta r=\sqrt{\frac{\sum_{t=1}^{N}{\left({\hat{C}}_{m,t}-C_{m,t}\right)}^{2}}{N-2}}$$

### 4.4. Real-Time Mass Concentration Modified by the Simultaneous Measured Particle Size

**Figure 14:**
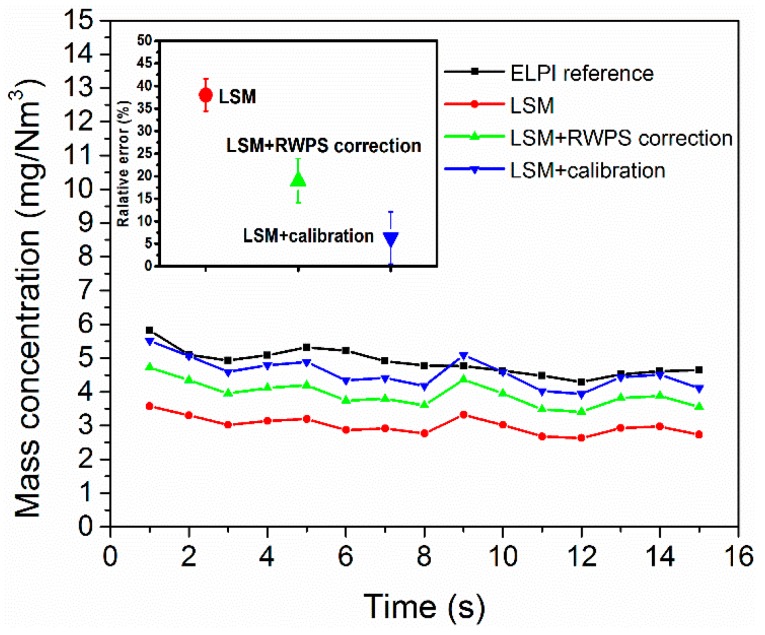
Comparison of real-time mass concentrations measured using the reference method (an electrical low pressure impactor (ELPI)), light scattering method (LSM), light scattering method combined with the ripple width particle size correction (LSM + RWPS) and light scattering calibrated with known particle (LSM + calibration).

According to the measured concentration of the four samples, the mass concentration measurement based on the light scattering method is affected by particle size. Therefore, the simultaneous measurement of particle size would be necessary to ensure the real-time accuracy of the concentration. As shown in Section 4.2, the particle size can be roughly measured in real-time within minutes using the ripple width method. The instantaneous nature of the particle size reading depends on the rotation period of the rotating detector, which provides an average ripple width and further particle size during one period. To show the correction of the ripple width particle size to the real-time particle concentration measurement, it was assumed that the aerosol size changed from B to C. According to the light scattering method (LSM), the relation between mass concentration and scattering light is reported as the relation of aerosol B, $C_{m}=3.78\times 10^{-3}*I(45{}^{\circ})+0.63$. Given that the light scattering method modified by the ripple width particle size (LSM+RWPS), the measured average particle size changed from 1.84 μm to 2.36 μm. To obtain more accurate correction, the aerosol size distribution is assumed to be the Rosin–Rammler distribution [58]. The dispersion index is set as 4.0, which matches best with the distribution measured from ELPI and the SEM images. Thus, the cumulative mass distribution can be described as $M(D)=1-\exp[-6.93\times 10^{-1}\times {\left(\frac{D}{D_{RWPS}}\right)}^{4.00}]$. Taking this information into consideration, the concentration transforming factor *K* can be calculated by Equation (6), and the result shows that *K_C_/K_B_* = 1.39. Thus, given the ripple width particle size, the relation has been amended as $C_{m}=5.25\times 10^{-3}*I(45{}^{\circ})+0.63$. The standard error ∆*r* of the measured mass concentration is 0.56 mg/Nm^3^ according to Equation (10). Based on these formulas, the real-time light intensity can be transformed into mass concentrations. Figure 14 presents the real-time mass concentrations of ELPI and the transform solutions. The LSM exhibits a large deviation from the reference measurement ELPI, which demonstrates that the concentration is inaccurate with the change of particle size, but with the correction from the ripple width particle size, the measurement deviation reduced obviously. The measurement error can be reduced from 38% to 18% with the real-time particle size measurement. And the results also indicate the measurement error of the light scattering method calibrated with known particles is ~6%, but it needs regular calibration, which is difficult for real-time calibration when particle size changes frequently. Thus, using the real-time ripple with particle size correction can effectively reduce the particulate matter mass concentration measurement error by light scattering method. 

The advantage of this method is using only two light detectors measuring the average particle size and particle mass concentration, which is more advanced than the traditional PM monitors without particle size information. With the measured particle size by this method, the measurement error of the real-time mass concentration is reduced obviously when particle size changed. This method is suitable for continuous monitoring of PM emission from coal-fired power plants.

The limitation of this method is that limiting the number of particles in the scattering volume to make the ripple method work would introduce a degree of uncertainty in estimating the actual ensemble particle size and concentration. Moreover, the best solution of using the real-time particle size to correct the mass concentration involves establishing a map, which contains the correlations of scattering light intensity with mass concentration calibrated for different particle size distributions or even different components. However, significant calibration work needs to be done, and this paper is limited to demonstrate this real-time particle size measurement method to improve the accuracy of particulate matter mass concentration monitoring. 

## 5. Conclusions

This work provides a new method to roughly measure the particle size from the ripple width of the scattering angular distribution in real time. The scattering patterns of quartz particles are calculated according to the Mie theory to study the relation between ripple and particle size. In addition, an aerosol optical measurement platform was generated to simultaneously measure the mass concentration and particle size of four quartz particles. The main conclusions are as follows:

1. The ripple widths of the scattering patterns for monodisperse aerosols are well fitted with the particle size by the power law.
2. Regarding moderate polydisperse aerosols, the scattering ripples can be washed out by the continuity of the size distribution. Nevertheless, given the artificial limit of the scattering volume, the particles in the scattering volume show discretely, and the scattering ripples can be reconstructed.
3. The particle size measured from the ripple width is compared with the particle size measured by ELPI. These measurements exhibit similar tendencies, and the relative error for the ripple width method compared with the ELPI result is less than 15%.
4. Particle size and mass concentration were simultaneously measured in our experimental setup, and the measurement error of real-time mass concentration is reduced from 38% to 18% with correction of the simultaneously measured particle size when the particle size has changed.

## Figures and Tables

**Table 1:** The slopes and the correlation coefficients of the linear fittings between the ripple width and particle size in Figure 2.

*D*/μm	0.5	1	2	3	4	5	6	7	8	9
Slope	63.9	30.6	19.5	13.8	11.3	8.1	7.0	5.9	5.3	4.6
R^2^	/	0.995	0.994	0.997	0.997	0.999	0.999	0.998	0.996	0.995

**Table 2:** The particle concentration and number of sample A with a size greater than 0.1 μm.

D_i_ (μm)	Number Concentration (cm^−3^)	Mass Concentration (mg/m^3^)	Particle Number
0.11	4432	0.01	69.59
0.18	1694	0.01	26.60
0.29	944	0.03	14.82
0.47	652	0.10	10.23
0.77	670	0.42	10.52
1.22	593	1.51	9.31
1.94	208	2.11	3.27
3.15	24	1.03	0.37
4.94	9	1.49	0.14
Total	9227	6.71	145

**Table 3:** Calculation parameters of particle size using the ripple width method.

Sample	Ripple Number *n*	Total Ripple Width ∑Δ*θ*/°	Average Ripple Width Δ*θ*/°	Particle Size *D*/μm	EPLI Measurements *D*/μm	Error
A	4	85°	21°	1.72	2.02	15%
B	4	80°	20°	1.84	2.03	9%
C	5	80°	16°	2.36	2.62	10%
D	5	75°	15°	2.54	2.63	3%
